# Novel Treatments for Diabetic Foot Osteomyelitis: A Narrative Review

**DOI:** 10.3390/microorganisms13071639

**Published:** 2025-07-11

**Authors:** Crystal Jing, Julia E. Ralph, Jamie Lim, Jackson M. Cathey, Conor N. O’Neill, Albert T. Anastasio

**Affiliations:** 1 School of Medicine, Duke University Health System, Duke University, 40 Duke Medicine Circle, Durham, NC 27710, USA; 2 Department of Orthopaedic Surgery, Duke University Health System, Duke University, 2301 Erwin Road, Durham, NC 27710, USA; conor.n.oneill@duke.edu

**Keywords:** diabetes, osteomyelitis, diabetic foot, therapy

## Abstract

Diabetic foot osteomyelitis (DFO) is a severe complication of diabetes mellitus and a leading cause of non-traumatic lower extremity amputation. Treatment remains clinically challenging with high recurrence rates despite standard antibiotic therapy and surgical debridement. This narrative review synthesizes current evidence on novel operative and nonoperative therapies for DFO, focusing on emerging biomaterials, local antibiotic delivery systems, innovative surgical techniques, and adjunctive topical agents. Studies examining bioabsorbable and nonabsorbable antibiotic carriers, such as calcium sulfate beads, collagen sponges, and bioactive glass, demonstrate promising infection resolution rates and a potential to reduce the surgical burden, though most are limited by small cohorts and observational designs. Similarly, alternative surgical approaches (i.e., cancelloplasty, conservative bone excision, and tibial cortex distraction) have shown early success in limb preservation. Nonoperative strategies, including adjunct antimicrobials, antimicrobial peptides, and topical oxygen, offer additional options, particularly for patients unfit for surgery. While initial outcomes are encouraging, the supporting evidence is heterogeneous and primarily limited to case series and small, noncomparative trials. Overall, these novel therapies show potential as adjuncts to established DFO management, but further prospective research is indicated to define their long-term efficacy, safety, and role in clinical practice.

## 1. Introduction

Diabetes affects over 537 million individuals globally, with diabetic foot ulcers affecting nearly 25% of patients; these ulcers may progress to more severe infections [1]. Normal wound healing consists of three phases: inflammation, proliferation, and remodeling; however, in patients with diabetic foot complications, this cycle of healing is disrupted, leading to difficulties with wound healing [2]. More specifically, patient with severe diabetes have compromises to their immune systems, in which pro-inflammatory M1 macrophages fail to transition to anti-inflammatory M2 macrophages, leading to disturbances in wound healing pathways [2].

A feared complication of progressive diabetes mellitus is foot infection due to insensate distal limb, with extensive soft tissue and skin involvement potentially progressing to diabetic foot osteomyelitis. These infections remain difficult to treat and are the most common complication of diabetes leading to lower extremity amputation (LEA) [3,4]. LEA is associated with markedly increased morbidity, with some studies suggesting risk profiles comparable to those seen in patients with colon, prostate, or breast cancer [5].

In patients with DFO, infections are most often localized to the forefoot, as diabetic foot ulcers frequently develop in this region and can lead to the contiguous spread of infection to the underlying bone [6]. There exist a variety of causative organisms of DFO, often dependent on geographic factors such as climate, environmental exposures, and chronicity of infection [7]. In warmer climates, isolated microorganisms are more likely aerobic gram-negative species, whereas in the Western hemisphere, gram-positive cocci, gram-negative bacilli, enterococci, and coagulase-negative staphylococci are more common [8,9]. Increasing rates of multi-drug resistant, non-fermenting, gram-negative rod DFO has rendered management of these infections particularly challenging [7,10].

Traditionally, DFO has been treated with medical management of the patient’s underlying diabetes, antibiotic therapy targeted to the causative microorganism, and local wound care with consideration for formal surgical management for more extensive debridement when necessary [11,12]. Despite these mainstays of treatment, DFO remains difficult to treat with high rates of morbidity and mortality [13,14].

Given the substantial rates of recurrence under traditional management guidelines, novel therapies for the treatment of DFO have garnered recent interest. Therapeutic approaches span surgical materials, novel surgical techniques, and nonsurgical options including topical and localized treatments. In this study, we reviewed the existing literature describing novel therapies for the treatment of DFO.

## 2. Materials and Methods

We conducted a narrative review, synthesizing available literature following a search query conducted on 1 June 2025. Databases, including MEDLINE via PubMed, Embase, and ClinicalTrials.gov, were queried to identify full-text articles discussing novel treatments for diabetic foot osteomyelitis. This study used the following search terms: “diabetic foot osteomyelitis” AND ((“novel treatment”) OR “treatment”). Following this search, 539 studies were identified. Studies were included in this review if they discussed management for DFO that differed from the mainstay treatments of antibiotics and/or surgical debridement and resection. No restrictions were placed on the publication date, original language of the published material, or study design. Our study included both preclinical and clinical research to best capture the range of novel treatments available. This review was supplemented with a brief discussion of novel treatments for the potential sequalae of DFO (i.e., abscess, soft tissue infection), given the prevalence of concomitant infection beyond the bone under traditional management [15].

## 3. Review

### 3.1. Intraoperative Therapies

There exist a variety of bioabsorbable and nonabsorbable therapies that may be utilized intra-operatively or in the peri-operative setting for patients with DFO. In addition, surgical techniques serving as an alternative to amputation have also been described (Table 1).

#### 3.1.1. Bioabsorbable Therapies

Novel antibiotic application techniques have gained traction in the treatment of DFO. Current literature reflects a growing interest in antibiotic-impregnated sponges and beads, which may enhance local antibiotic delivery to infection sites while minimizing the adverse effects of intravenous antibiotics at therapeutic levels [16,17]. Bioabsorbable sponges and beads are particularly promising as they limit the increased infection risk of subsequent removal procedures required by polymethylmethacrylate (PMMA) beads, which have been found to facilitate bacterial growth themselves upon culture [18,19]. Krause et al. described in a retrospective comparative study of 65 feet that revision rates significantly decreased in patients receiving tobramycin-impregnated calcium sulphate beads during transmetatarsal amputation (TMA) as compared to no beads during TMA (8.2% versus 25%) [20]. While limited by a small sample size, their study demonstrated promising results and offered a viable single-stage option for medically complex patients [20].

Gauland et al. conducted a cohort study of 323 patients receiving antibiotic-impregnated calcium sulphate beads in the general treatment of lower extremity osteomyelitis. This study reported that 86.4% (*n* = 279/323) of patients demonstrated clinical resolution following surgical debridement and bead implantation without the use of intravenous antibiotics. This study population included a subset of patients with compromised perfusion to the lower extremity (defined as an ankle-brachial index < 0.7 and/or transcutaneous oxygen readings < 40 mmHg); although ischemic disease was an independent risk factor for poor osteomyelitis recovery, these patients still demonstrated successful healing [21,22]. Most patients received vancomycin and gentamicin in synthetic calcium sulfate, though alternatives like cefazolin or gentamicin/cefazolin were used when clinically indicated or due to allergies. No differences in wound drainage were observed between antibiotic combinations [21].

In a retrospective comparative study of 48 limbs (46 patients), Qin et al. reported on outcomes of DFO patients undergoing infected bone resection with vancomycin and/or gentamicin-impregnated calcium sulfate as compared to bone resection alone [23]. They found that 90% (*n* = 18/20) of limbs in the calcium sulfate group achieved full recovery as compared to 78.6% (*n* = 22/28) of limbs in the control group; however, this difference did not reach statistical significance [23]. Patients who received calcium sulfate did not experience any recurrence of osteomyelitis, while 36.4% (*n* = 8/22) of the control group developed recurrent osteomyelitis; this difference was found to be statistically significant [23]. They also reported no significant differences in the time to wound healing, amputation rate, and postoperative leakage between the two cohorts [23]. Further investigation of larger patient cohorts will be integral in characterizing the potential benefits conferred by calcium sulfate beads.

**Figure 1:**
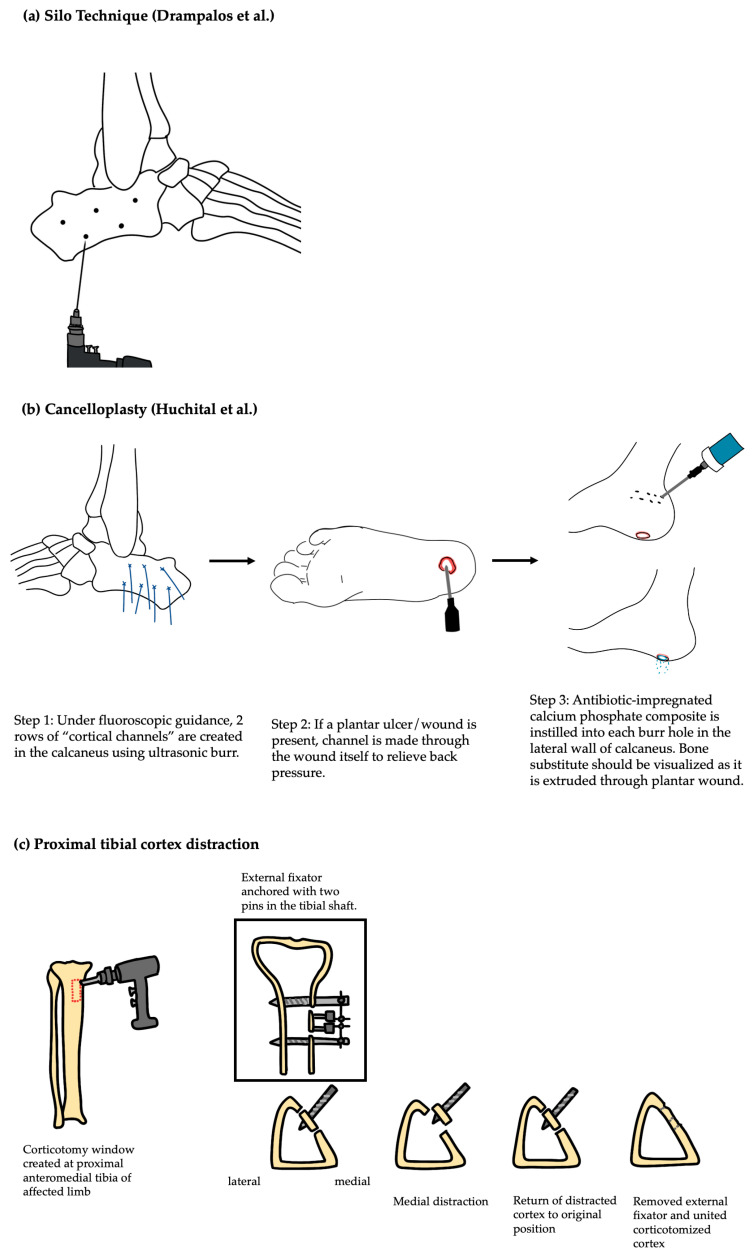
Schematic of novel surgical techniques. Reference to respective articles [25,40].

Hybrid calcium sulfate and hydroxyapatite (CS/HA) bio-composites have emerged as viable options to treat osteomyelitis due to hydroxyapatite’s similar composition to natural bone [24]. Drampalos et al. described the Silo surgical technique for the treatment of calcaneal DFO, involving the debridement of devitalized bone and local delivery of CS/HA–antibiotic mixtures via multiple drilled tunnels in the calcaneus (Figure 1a) [25]. Drampalos reported on 12 consecutive diabetic patients with heel ulcers and calcaneal osteomyelitis undergoing treatment. The most common causative organisms affecting this study population were *Staphylococcus aureus* (*n* = 4), *Escherichia coli* (*n* = 3), and *Pseudomonas aeruginosa* (*n* = 2). Infection was eradicated in all patients at a mean 16 weeks postoperatively [25]. Niazi et al. conducted a multicenter retrospective cohort study on a similar intervention, utilizing debridement and the local delivery of CS/HA–antibiotic mixtures in 70 patients, predominantly infected with *Staphylococcus aureus*, with Texas Grade 3B and 3D lesions [26]. The authors reported favorable outcomes, with infection eradicated in 90% (*n* = 63/70) of patients and ulcer resolution occurring at a mean time of 12 weeks following treatment [26].

As calcium sulfate beads demonstrate promising results in treating DFO, alternative devices, such as sponges, have displayed similar outcomes. Gentamicin-impregnated collagen sponges, specifically Collatamp^®^ (Syntacoll GmbH Saal, Saal an der Donau, Germany), have demonstrated a reduced wound infection risk in general and cardiac surgery [27]. The Collatamp^®^ implant contains of bovine collagen and gentamicin sulfate, with human collagenase degrading the implant and allowing for the active diffusion of gentamicin to the infection site [28,29]. Varga et al., in a prospective, randomized trial studying the efficacy of Collatamp^®^, found a significantly shortened time to wound healing, at approximately 2 weeks, in patients receiving amputation and Collatamp^®^ versus those undergoing amputation without local antibiotics [30]. The authors did not report any statistically significant differences in the length of the hospital stay, number of revisions for wound breakdown, or number of re-amputations [30].

The novel biomaterial S_53_P_4_ bioactive glass (BG) has become of interest in the treatment of DFO. BG was first invented by Lerry Hench at the University of Florida in 1969 [31] and has remained of particular interest due to its unique antimicrobial, osteo-stimulative, and osteoconductive properties [32]. Recent work has focused on the use of S_53_P_4_ BG in the general setting of osteomyelitis as an agent to fill voids following debridement [33]. Evidence supporting the use of S_53_P_4_ BG remains limited compared to previously described therapies for DFO [6]. Iacopi et al. conducted a pilot study, in which 10 patients with DFO were implanted with BG during surgical debridement [34]. They found that one patient required a second surgery; no patients had reported recurrence or complications in the follow-up period [34]. De Giglio et al. conducted an observational retrospective study of 22 patients with DFO implanted with BG following surgical debridement and compared their outcomes to 22 consecutive controls that underwent debridement only, with all patients receiving systematic antibiotics [32]. Patients with BG implantation achieved infection eradication at a significantly higher rate than the controls (90%, *n* = 18/22 versus 61.9%, *n* = 13/22).

In a retrospective cohort study of 22 patients undergoing segmental resection of the first metatarsophalangeal joint by Kastrin et al., they compared a group of patients receiving gentamicin sulfate-impregnated beads (*n* = 12) with a group of patients receiving BG (*n* = 10) [35]. At the end of the follow-up, 75% (*n* = 9/12) of patients receiving beads experienced the full eradication of infection; one patient who did not recover developed methicillin-resistant *Staphylococcus aureus* (MRSA) in the postoperative period [35]. All 10 patients receiving BG experienced complete eradication of infection [35].

#### 3.1.2. Nonabsorbable Therapies

Nonabsorbable therapies offer relative advantages in the durability and prolonged delivery of antibiotics. One such nonabsorbable device is an antibiotic-impregnated cement spacer (ACS). Recent literature reflects growing interest in the utilization of ACS in DFO treatment [36,37]. Melamed et al. reported on a case series of 23 patients with severe DFO treated with ACS and intensive surgical debridement [38]. They found that 91.3% (*n* = 21/23) of patients healed at the final follow-up and 8.7% (*n* = 2/23) required subsequent toe amputation [38]. The authors noted that ACS was particularly helpful in filling large voids, allowing for more extensive tissue debridement [38]. Khury et al. conducted a retrospective review of 55 DFO patients treated with ACS and found that 57.72% (*n* = 29/55) achieved full eradication of infection without further intervention and 14.54% (*n* = 8/55) required an additional procedure to achieve complete eradication [39]. They found that significant risk factors for reoperation included age, gender, and use of gentamicin-only ACS [39].

#### 3.1.3. Surgical Technique

There are surgical techniques described in the literature that propose alternatives to amputation or more invasive procedures for severe cases of DFO. Huchital et al. presented a case report describing a novel technique for calcaneal osteomyelitis: cancelloplasty with a vancomycin- and gentamicin-impregnated calcium phosphate bone substitute delivered through burr holes in the calcaneus (Figure 1b). The authors described a patient who was infected with gentamicin-sensitive *Pseudomonas aeruginosa* [40]. Postoperatively, the patient’s wound depth decreased, and topical xenografts were used to epithelize the superficial aspect of the wound [40]. No additional operations were required. At the final follow-up, the patient was able to ambulate independently and returned to work without restriction [40]. Lázaro-Martínez et al. reviewed seven types of conservative surgical techniques for DFO of the forefoot, including partial or total distal phalangectomy, arthroplasty of the proximal or distal interphalangeal joint, distal Syme amputation, percutaneous flexor tenotomy, arthroplasty of the metatarsophalangeal joint, sesamoidectomy, and metatarsal head resection [41]. These forms of conservative surgeries have been shown to be safe and effective in wound-healing, infection control, and decreasing mortality [42,43].

Conservative approaches to surgery involving minimal local resections have also been applied to decrease the morbidity involved with invasive approaches such as LEA in patients with DFO. Freeman et al. described a novel surgical technique with metatarsal excision in seven patients with Grade III Wagner classification lesions. Pre-operative levels of mobility were achieved in 71.4% (*n* = 5/7) of patients at a mean of 12.6 days. Although 28.5% (*n* = 2/7) of patients were readmitted with wound infection within the 30-day postoperative period, no patient in the cohort required amputation [44]. Likewise, Schöni et al. conducted a retrospective comparative effectiveness study with 651 patients, comparing conservative surgery *(n* = 121), defined as the local excision of infected bone and devitalized tissue, to amputation (*n* = 530) [45]. The authors found that patients undergoing conservative surgical approaches experienced significantly increased rates of revision as compared to those with minor amputations [45]. Percutaneous partial bone excision has also been described as a potential alternative to amputation. Moosa et al. conducted a prospective cohort study of 47 patients receiving percutaneous partial bone excision for DFO of the toes [46]. The authors found that 93.6% (*n* = 44/47) of patients experienced full wound healing, with just 6.4% (*n* = 3/47) requiring subsequent toe amputation. A subset of patients experienced persistent clinical infection (25.5% [*n* = 12/47]); nine of those patients exhibited positive culture growth and one patient with MRSA required subsequent amputation [46].

Proximal tibial cortex transverse distraction is another novel surgical technique that has gained recent traction in the literature (Figure 1c). The longitudinal distraction of the proximal tibia allows the stimulation of vascularization and tissue regeneration in the distal tibia, offering promise in patient populations with compromised perfusion and distal wound-healing issues, such as those affected by diabetes mellitus [47]. This technique utilizes external fixator devices, thus not requiring specialize equipment or technology [48]. Chen et al. described the use of this surgical technique in a therapeutic study of 136 patients for recalcitrant diabetic foot ulcers to promote the vascularization and regeneration of surrounding tissue to prevent the development of DFO. At the end of the two-year follow-up period, patients undergoing tibial cortex transverse distraction had a greater proportion of healed ulcers as compared to the control group, who underwent traditional surgical debridement [49]. Jianda et al. also described a similar technique of longitudinal distraction of the proximal medial tibia for the treatment of calcaneal osteomyelitis in a retrospective cohort study. The five patients with calcaneal osteomyelitis included in this study exhibited a mean time to wound healing of 141.2 ± 19.1 days. No patient required amputation [50]. Although transverse distraction has shown early promise, evidence remains limited. Future studies with larger cohorts with longer follow-up times will be integral in determining the efficacy of this procedure and optimizing patient selection criteria.

### 3.2. Nonsurgical Therapy

Various nonsurgical DFO treatments have emerged with the goal of minimizing invasive surgical interventions while preserving limb integrity and leveraging local administration to deliver high concentrations of therapeutic agents directly to affected tissues (Table 2) [51].

#### 3.2.1. Adjunct Therapy to Antibiotics

Traditional nonsurgical management includes a course of systemic antibiotics; novel therapies have been studied as adjuncts to systemic regimens. Two retrospective studies by Wilson et al. and Senneville et al. report on the addition of rifampin to antimicrobial regimens improved eradication rates for DFO [52,53]. There is currently a Phase 4 clinical trial (NCT03012529) investigating the role of rifampin in the treatment of DFO; this trial spans a six-week course observing adjunctive rifampin versus adjunctive matched placebo (riboflavin) in combination with mainstay antimicrobial therapy [54]. In addition, antimicrobial peptides (AMPs), innate immune system molecules that serve as a natural first line of defense, have garnered increasing interest in antimicrobial research and may serve as potential adjuncts for common microorganisms observed in DFO [55]. These AMPs are of particular interest in combatting antibiotic-resistant bacteria. Goldberg et al. conducted an in vivo study reporting on a novel AMP, C12(ω7)K-β12, that temporarily depolarizes cell membranes, disrupting the proton-motive force required by bacterial antibiotic efflux pumps and thus re-resensitizing previously resistant strains [56]. Vargas et al. demonstrated that the application of a peptide sequence derived from naturally-occurring AMPs stopped bacterial growth in the biofilm of MRSA-infected wounds in TALLYHO mice [57]. Gupta et al. reported on the ability of cholic acid–peptide conjugates, a type of AMP, to degrade single-species and polymicrobial biofilms of *Staphylococcus aureus* and *Candida albicans* [58]. Other preclinical studies have demonstrated BP203 and MAP-0403 J-2 as potential AMPs against colistin-resistant *E.coli* and *K. pneumoniae*; SR-25 has also demonstrated potential against *E. coli* and MRSA [59,60]. However, the efficacy of AMPs has not been well-established in clinical practice.

There is a current Phase 3 interventional clinical trial (NCT05074147) comparing the efficacy and tolerance of 3 versus 6 weeks of antibiotic therapy [61]. This study was driven by the increase in multi-drug-resistant organisms, such as MRSA, and assesses if a shortened antibiotic course can effectively treat DFO.

#### 3.2.2. Topical and Local Therapy

In a randomized cohort study of 50 diabetic patients following minor amputation for nonhealing ulcers with osteomyelitis, Varga et al. found that the use of a gentamicin–collagen sponge with systemic antibiotics shortened the wound healing duration by nearly two weeks compared to those who only received systemic antibiotics [30].

In the case of diabetic foot abscesses, a feared DFO complication, current standards of care involve immediate surgical drainage and debridement [62]. As this may result in some extent of minor amputation and bony loss, conservative treatments for diabetic foot abscesses have been explored to avoid more invasive procedures. Cahn et al. presented a case series of patients with diabetic foot abscess and osteomyelitis who were treated with topical oxygen and had abscesses drained using PolyMem^®^ Wic^®^ Silver Rope (PWSR) (Ferris Mfg., Fort Worth, TX, USA) twice a week for a period of time ranging from 3 to 8 months [63]. Patients were treated with topical oxygen using TOPOX, a device that supplies topical oxygen to the foot wound surface via an extremity chamber [63]. Topical oxygen promotes wound healing by activating phagocytosis, increasing reactive oxygen species levels in the wound environment, and promoting angiogenesis in the wound edge tissue [64]. All patients who received topical oxygen and abscess drainage with PWSR achieved complete recovery within 2–9 months and no recurrence of their wounds or osteomyelitis during a 4 to 21 month follow-up period. Though this case series concluded that nonsurgical treatment using topical oxygen and PWSR is an acceptable therapeutic method, larger studies are needed to validate these findings [63]. A prospective study by Blackman et al. reported the efficacy of topical oxygen compared to silver-based dressing in the treatment of severe diabetic foot ulcers [65]. Wounds treated with topical oxygen had a significantly higher likelihood of healing over shorter periods of time compared to wounds treated with the silver-based dressing. However, there was no formal randomization conducted in this study, again demonstrating the need for larger, randomized trials to confirm the efficacy of this therapeutic approach. Although systemic hyperbaric oxygen therapy is well-established in wound and ulcer healing, particularly for diabetic foot ulcers refractory to treatment, there have been no studies on the effect of hyperbaric oxygen therapy directly treating the infectious aspect of DFO [66]. Topical oxygen therapy presents several advantages over systemic hyperbaric oxygen therapy, including greater accessibility for patients, lower costs, and a lack of systemic oxygen toxicity risks [67].

## 4. Discussion

In our review, we provide a broad overview of existing literature on novel therapies and surgical techniques for the treatment of DFO. Current published reviews focus on broader diabetic foot infections or more focused traditional treatments and adjuncts [68]. We uniquely survey both preclinical and clinical studies to capture all ongoing research on DFO management.

### 4.1. Critical Comparison of Treatment Types

In this study, we broadly overviewed surgical and nonsurgical treatment therapies for DFO. Surgical interventions with the greatest body of literature involve the use of bioabsorbable products during surgical debridement, specifically antibiotic-impregnated calcium sulfate beads and calcium sulfate hydroxyapatite bio-composites. Across the three studies, including over 380 patients, analyzing antibiotic-impregnated calcium sulfate beads, clinical resolution was observed in 75% to 90% of patients without any reported cases of recurrence during study follow-up periods [21,23,35]. Bioactive glass has fewer existing data, with a total of 42 patients across the three studies reviewed; however, there is an ongoing clinical trial assessing its efficacy in DFO. The resolution of DFO following the implantation of BG during surgical debridement was reported to range from 80% to 100% [32,34,35]. Novel surgical techniques, focused on conservative excision, have been proposed as an alternative to amputation. Cancelloplasty was described in a case report, demonstrating good outcomes without reoperations [40]. Metatarsal excision was described in a small cohort of patients, with 71.4% achieving pre-operative levels of mobility, 28.5% readmitted for wound infection within the 30-day postoperative period, and no patients undergoing subsequent amputations [44]. The local excision of infected bone has demonstrated poorer outcomes, with one study reporting significantly higher rates of revision in these patients as compared to amputation patients [45] and another study reporting 25.5% of continued infection with 6.4% of patients going on to receive an amputation [46]. Current data on nonsurgical therapy remain in the preliminary stages of research, with ongoing clinical trials and in vivo studies.

### 4.2. Clinical Indications and Guidance

There exist many growing avenues for the development of novel treatments for DFO. Patients may be suitable for conservative surgical approaches when they have a localized infection or necrosis where limb preservation is feasible. The Infectious Diseases Society of America (IDSA) and the International Working Group on the Diabetic Foot (IWGDF) recommended the consideration of conservative surgical resection when there is exposed bone, undrained abscesses, signs of compartment syndrome, or a severe infection that cannot be managed with antibiotics alone [62]. In addition, these surgical approaches as compared to amputation may reduce morbidity, which is an important consideration in patients with significant cardiac or systemic comorbidities [41].

As there are many conservative surgical techniques, optimal patient selection is important. Patients with localized bone defects are the most appropriate surgical candidates, as they carry a lower risk of requiring subsequent revision surgeries. The IWGDF/IDSA guidelines recommend conservative surgical intervention in patients with forefoot DFO, without extensive soft tissue damage or ischemia [45,62,69]. Moreover, patients with vascular disease may require revascularization procedures prior to conservative surgery to optimize post-operative wound healing [62].

For patients not deemed appropriate surgical candidates, rifampin may be considered as an adjunct to antimicrobial therapy for a more aggressive approach to infection eradication, as current studies have demonstrated improved infection clearance [52,53]. Thus, rifampin has demonstrated the greatest efficacy of all novel nonsurgical treatments discussed in our presents study [52,53].

### 4.3. Economic Impact

Diabetic foot osteomyelitis carries significant health and economic burdens, with inpatient hospital costs, long-term antibiotic therapy, and the frequent need for multiple surgical operations [70]. Krause et al. utilized OsteoSet-T^TM^ (Wright Medical Technologies, Arlington, TN, USA) calcium sulphate antibiotic beads and Qin et al. utilized Stimulan calcium sulphate beads (Biocomposite Ltd., Keele, UK) [20,23]; these implants and similar products have been reported to range from USD 400 to 500 per patient [71]. The study by Chia et al. utilized a Collatamp G (Schering-Plough, Stockholm, Sweden) gentamicin–collagen sponge, costing USD 180 to 360 per patient depending on the size of the implanted sheet; Varga et al. studied a similar product [27,30]. Niazi et al. utilized CERAMENT G, a bone void filler [26]; although not reported in their study, literature on the cost-effectiveness of this product has been estimated to be up to EUR 4000 (USD 4712) per patient for chronic osteomyelitis [72]. Other novel therapies, such as BG S53P4, have listed market prices starting from USD 451 per 10 g [73]. Guerts et al. compared the total health care costs between BG and PMMA treatment cohorts, reporting significantly lower health care costs in the BG-treated patients (EUR 6573 vs. 20,568.31, respectively). Similarly, with adjunct intra-operative materials and the need for additional imaging, material costs increased significantly with BG, as compared to the PMMA cohort (EUR 1640, 95% CI: EUR 1123–2256 for BG implant per patient and EUR 817, 95% CI: EUR 208–1368 for additional imaging) [74]. Despite this study by Guerts et al. demonstrating decreased total health care costs with the use of PMMA, it must be taken into consideration that this nonabsorbable material may require subsequent procedures, thus increasing future costs and morbidity.

Chan et al. generated a model to predict the cost-effectiveness of diffusion oxygen therapy for patients with diabetic foot ulcers, reporting that in comparison to negative wound pressure therapy, topical oxygen would cost USD 4800 less per patient [75].

The use of therapeutic adjuncts requires increased operating room (OR) or inpatient hospital costs; however, some argue that the low-profile side effects and promising efficacy of these therapies may reduce future hospitalizations and surgeries to minimize downstream healthcare costs. Monami et al. conducted a cost analysis and demonstrated in their study that patients receiving calcium sulphate granules as compared to those treated with traditional approaches did not have differences in direct costs [76]. Serrier et al. conducted cost analyses and reported that hospitalization for chronic osteomyelitis could range from EUR 5518 to 19,608 and subsequent amputations costing from EUR 5130 to 18,481 [72]. In addition, due to the known difficulties with wound healing in patients with symptomatic diabetes, the ability to rehabilitate following surgery is an important consideration for DFO patients.

Lastly, the duration of systematic antibiotic therapy has both direct and indirect cost impacts. The IWGDF/IDSA guidelines suggest that an antibiotic duration of 6 weeks is adequate and longer courses have not been shown to provide additional benefits [62]. Thus, shorter antibiotic courses are less costly and have a decreased risk of antibiotic resistance [77]. One Australian study estimated that the median cost of antibiotics for their diabetic foot infection cohort was AUD 969 per patient, with intravenous antibiotics costing over 100-fold more than oral antibiotics [78].

### 4.4. Postoperative Quality of Life

Clarke et al. generated a model to evaluate the impact on the quality of life of amputation on patients with diabetes, reporting a tobit model tariff value of −0.280 (95% CI = −0.389, −0.170), indicating that amputation negatively limits the postoperative quality of life [79]. The novel therapies discussed in our review aim to prevent amputations. Momani et al. performed a randomized control trial analyzing the effect of calcium sulphate antibiotic delivery compared to a placebo treatment; they found that Short-form-12 (SF-12, a questionaries series to assess physical and mental well-being) scores were significantly better in the calcium sulphate group as compared to controls [76]. Chan et al. conducted a quality-adjusted life years analysis on the use of topical oxygen on diabetic foot ulcers, reporting an increase of 0.025 as compared to traditional negative pressure wound therapy [75].

The impact on the quality of life for conservative surgery is more controversial, with Schöni et al. reporting that although other clinical outcomes and infection recurrences were similar to amputation patients, there were significantly more revision surgeries in the conservative surgery group [45].

### 4.5. Technical Barriers

With novel therapies comes a learning curve. There exists limited literature discussing technical barriers of conservative and alternative surgical techniques. However, it is well known that antibiotic cement delivery techniques are important in the efficacy and clinical outcomes for patients [80].

S53P4 BG may pose challenges, as the granular consistency can be difficult to mold into bone defects. Difficulties with contouring may lead to challenges with filling dead space, infection control, and complications with the material. However, Nguyen et al. conducted a systematic review on the use of S53P4 BG in osteomyelitis and reported rare cases of migration and low rates of infection recurrence and reoperations [81]. Gatti et al. suggested that to optimize the effectiveness of S53P4 BG in bone void filling, autologous bone grafts should be utilized in conjunction with BG for volumes greater than 10–15 mL [82].

### 4.6. Limitations

Limitations of this review include the variability across studies. Clinical resolution is defined differently across each study, and there exists a wide range of outcomes assessed. In addition, we were unable to conduct qualitative analyses to compare studies for similar reasons.

## 5. Conclusions

DFO remains a challenging complication of diabetes, posing a significant risk of progression to limb-threatening infection and amputation. While traditional approaches (i.e., systemic antibiotics, glycemic control, surgical debridement) form the foundation of care, high recurrence rates underscore the need for improved therapeutic strategies. This narrative review highlights a host of novel interventions, including bioabsorbable antibiotic carriers, nonabsorbable cement spacers, advanced surgical techniques, and emerging nonsurgical modalities such as antimicrobial peptides, topical oxygen therapy, and medicated dressings. Early outcomes from these therapies are promising, particularly in enhancing local antimicrobial delivery and limb preservation. Despite these results, current evidence remains limited by small sample sizes, heterogeneity in study design, and short follow-up durations. Further large-scale, prospective studies are needed to evaluate the efficacy, safety, and optimal clinical indications for these innovations. Until a growing body of level I and II evidence demonstrates concordance in the utility of these agents as monotherapy for DFO, these therapies should be used as adjuncts to standard care rather than definitive replacements.

## Figures and Tables

**Table 1:** Summary of intraoperative therapies.

Intervention	LOE	Mechanism	Clinical Outcomes	Limitations
Bioabsorbable Therapy				
Calcium sulfate beads	III, IV (therapeutic)	Antibiotic-impregnated calcium sulfate beads provide high local antibiotic levels without systemic toxicity and gradually dissolve, eliminating removal while filling dead space and controlling infection in poorly vascularized bone.	Across three key studies of over 380 patients treated with antibiotic-impregnated calcium sulfate beads, clinical resolution was reported in 75% to 90% of cases with no recurrences during follow-up.	Bead implantation remains an invasive adjunct requiring surgical placement, which may not be suitable for all patients.
Collagen-based implants	IIa (therapeutic), preclinical	Collagenase degrades the collagen matrix, enabling sustained local gentamicin release directly to the infection site while the implant is resorbed.	Gentamicin-impregnated collagen sponges reduce wound infection risk and speed healing in DFO, with one trial reporting ~2-week healing post-amputation, without affecting hospital stay, revision, or re-amputation rates.	Collagen sponges require surgical placement and may not address dead space as effectively as other local delivery systems.
Bioactive glass	IIb, IV (therapeutic), preclinical	BG provides antimicrobial activity through local pH elevation and ion release, while supporting bone regeneration via osteostimulation and osteoconduction.	BG has demonstrated infection eradication rates of 90% to 100% in small DFO cohorts, with no reported recurrences and superior outcomes compared to debridement alone.	BG requires surgical implantation, may be challenging to contour for complex defects, and its resorption rate can be unpredictable, potentially affecting bone healing.
Nonabsorbable Therapy				
Antibiotic-impregnated cement spacer	IV (therapeutic)	ACS provides prolonged local antibiotic delivery while filling dead space and offering temporary structural support following debridement.	ACS has shown infection eradication rates of 58% to 91% in small DFO case series, with some patients requiring additional procedures or amputations.	ACS is nonabsorbable, requiring removal or revision, and poses a risk of biofilm formation if retained; shaping can be challenging in small or irregular defects.
Surgical Technique				
Conservative bone-sparing techniques	IIb, III, IV (therapeutic)	These techniques aim to limit infection through selective removal of infected bone while preserving limb structure and function.	Conservative techniques like metatarsal excision and partial bone removal show mixed outcomes, with good mobility or healing in most cases but notable risks of infection, readmission, and higher revision rates compared to minor amputation.	Outcomes across and within techniques are inconsistent and are further associated with risks of persistent infection, wound complications, and need for revision.
Adjunctive regenerative techniques	III, IV (therapeutic)	Cancelloplasty fills dead space with antibiotic-loaded bone substitute to eradicate infection; tibial distraction promotes angiogenesis and tissue regeneration through controlled mechanical strain.	Adjunctive techniques like cancelloplasty and tibial distraction show promise in managing diabetic foot osteomyelitis, with early reports noting full healing without reoperation in isolated cases and improved ulcer healing and wound closure rates without amputation in small series.	Such techniques require specialized expertise and equipment, with risks of pin-site infection (distraction) and limited generalizability.
Conservative bone-sparing techniques	IIb, III, IV (therapeutic)	These techniques aim to limit infection through selective removal of infected bone while preserving limb structure and function.	Conservative techniques like metatarsal excision and partial bone removal show mixed outcomes, with good mobility or healing in most cases but notable risks of infection, readmission, and higher revision rates compared to minor amputation.	Outcomes across and within techniques are inconsistent and are further associated with risks of persistent infection, wound complications, and need for revision.

DFO: diabetic foot osteomyelitis; BG; bioactive glass; ACS: antibiotic-impregnated cement spacer; LOE: level of evidence.

**Table 2:** Summary of nonsurgical management therapies.

Intervention	LOE	Mechanism	Clinical Outcomes	Limitations
Adjunct therapy to traditional antibiotic regimens				
Rifampicin	I (ongoing), III (therapeutic)	Rifampicin provides broad-spectrum antimicrobial activity with strong bone penetration and biofilm-disrupting properties, enhancing bacterial eradication in osteomyelitis.	Retrospective studies report that adding rifampicin to standard antimicrobial regimens improves DFO eradication rates. An ongoing clinical trial is investigating outcomes of adjunctive rifampicin versus placebo.	Rifampicin can be hepatotoxic; may also introduce drug interactions with concomitant use of medications metabolized by CYP3A4.
Antimicrobial peptides	preclinical	AMPs target bacterial membranes, causing depolarization and disruption of the proton-motive force, which impairs efflux pump function and enhances susceptibility to antibiotics.	Data on AMPs in DFO are limited to preclinical studies; in vivo models have demonstrated the ability to resensitize antibiotic-resistant bacteria by disrupting bacterial efflux mechanisms.	Evidence is limited to preclinical studies without human data; foreseeable challenges include stability, delivery, and potential cytotoxicity.
Topical and local therapy				
Topical oxygen therapy	IIb, IV (therapeutic)	Topical oxygen promotes phagocytosis, increases reactive oxygen species, and stimulates angiogenesis, supporting wound healing.	Case series report complete recovery in patients with diabetic foot abscesses treated with topical oxygen and drainage, with no recurrences overlong-term follow-up. A prospective study found higher healing rates in diabetic foot ulcers treated with topical oxygen compared to silver-based dressings.	Evidence focuses on abscess and ulcer healing, with limited utility for directly treating underlying DFO. Topical oxygen therapy on an additional medium for sustained delivery.
Medicated wound dressing	IIb, IV (therapeutic)	Silver-impregnated dressing provides sustained antimicrobial activity within the wound bed while facilitating drainage.	Case series using PWSR with drainage in diabetic foot abscesses reported complete recovery within 2–9 months and no recurrence during follow-up.	Wound dressings exhibit limited utility in treating underlying DFO, silver presents concern as potential topical irritant.

DFO: diabetic foot osteomyelitis; Cytochrome: P450 3A4; AMP: antimicrobial peptide; PWSR: PolyMem^®^ Wic^®^ Silver Rope.

## Data Availability

No new data were generated or analyzed in this study. All information presented is based on previously published literature, which is cited accordingly in the manuscript.

## Abbreviations

The following abbreviations are used in this manuscript:

DFO	Diabetic foot osteomyelitis
LEA	Lower extremity amputation
MRSA	Methicillin-resistant *Staphylococcus aureus*
TMA	Transmetatarsal amputation
